# Cosavirus Infection in Persons with and without Gastroenteritis, Brazil

**DOI:** 10.3201/eid1804.111415

**Published:** 2012-04

**Authors:** Andreas Stöcker, Breno Frederico de Carvalho Dominguez Souza, Tereza Cristina Medrado Ribeiro, Eduardo Martins Netto, Luciana Oliveira Araujo, Jefferson Ivan Corrêa, Patrícia Silva Almeida, Angela Peixoto de Mattos, Hugo da Costa Ribeiro, Diana Brasil Pedral-Sampaio, Christian Drosten, Jan Felix Drexler

**Affiliations:** University of Bonn Medical Centre, Bonn, Germany (A. Stöcker, C. Drosten, J.F. Drexler);; Federal University of Bahia, Salvador, Brazil (A. Stocker, B.F.C.D. Souza, T.C.M. Ribeiro, E.M. Netto, L.O. Araujo, J.I. Corrêa, P.S. Almeida, A.P. de Mattos, H.C. Ribeiro Jr, D.B. Pedral Sampaio)

**Keywords:** Picornaviridae infections, human cosavirus, Brazil, communicable diseases, virus shedding, gastroenteritis, pathogenesis, viruses

## Abstract

To determine possible cosavirus association with clinical disease, we used real-time reverse transcription PCR to test children and HIV-positive adults in Brazil with and without gastroenteritis. Thirteen (3.6%) of 359 children with gastroenteritis tested positive, as did 69 (33.8%) of 204 controls. Low prevalence, frequent viral co-infections, and low fecal cosavirus RNA concentrations argue against human pathogenicity.

The family *Picornaviridae* comprises 12 genera and includes several leading pathogens affecting human and animal health, e.g., foot-and-mouth-disease virus and polioviruses. With the advent of metagenomics, 3 novel human picornaviruses have been described since 2008: klassevirus, salivirus, and cosavirus (*1**–**3*).

Besides being detected in raw sewage (*4*), cosaviruses have been detected in human fecal specimens and were tentatively associated with gastroenteritis in 2 patients from Australia and Scotland (*3**,**5*). Cosavirus pathogenicity in humans has remained unknown, however, because detection rates in patients and healthy controls were similar in the only available cohort studies of patients with acute flaccid paralysis in Southeast Asia (*3*) and with gastroenteritis in China (*6*). We investigated cosavirus prevalence in 464 children and HIV-infected adults with gastroenteritis and 253 controls without gastroenteritis in Brazil.

## The Study

Fecal specimens from 6 clinical cohorts in Salvador, northeastern Brazil, were analyzed (Table 1). Adult cohorts comprised HIV-infected patients with gastroenteritis (105 persons) and without (49 persons) gastroenteritis. Child cohorts included 359 children with gastroenteritis and 204 healthy children from child-care centers**.** Nasal swab specimens were obtained from controls attending child-care centers and from children with gastroenteritis and concomitant respiratory symptoms. All specimens were stored at −30° to −80°C until further processing.

**Table 1:** Clinical cohorts tested for cosavirus, Salvador, Brazil*

Cohort no.	Cohort description†	Sampling site‡	Sampling time	Participant age, mo, mean (SD)	No. participants	No. (%) RT-PCR positive§	Virus concentration, log_10_ RNA copies/g feces, mean (SD)¶
1	HIV-infected adults with gastroenteritis	Infectious Diseases HIV Outpatient Department	2007 Mar–2010 Mar	487.6 (114.4)	105	1 (1.0)	4.43
2	HIV-infected adults without gastroenteritis		2007 Mar–2010 Mar	533.8 (115.9)	49	0	–
3	Children with gastroenteritis	Department of Pediatrics Metabolic Unit#	2006 Feb–2007 Sep	19.0 (15.6)	359	13 (3.6)	3.40 (0.93)
4	Control children without gastroenteritis	Community child-care center**	2008 Dec	29.6 (13.1)	132	65 (49.2)	2.97 (0.97)
5	Control children without gastroenteritis	Community child-care center**	2011 Nov–2011 Dec	14.3 (5.5)	62	4 (6.5)	3.41 (0.49)
6	Control children without gastroenteritis	University child-care center**	2011 Oct–2011 Nov	18.6 (4.2)	10	0	–
Total					717	83 (11.6)	

*RT-PCR, reverse transcription PCR; –, no virus obtained. †Gastroenteritis was defined as acute diarrhea with >3 watery stools in the previous 24 h and within 13 d before hospital admission. ‡All hospital units were located within the Hospital Professor Edgard Santos, Federal University of Bahia. All sites were located in Salvador, Bahia, in northeastern Brazil. §Samples only considered if positive in nested RT-PCR as in (*3*) and in strain-specific real-time RT-PCR. ¶Measured by strain-specific RT-PCR. #Reports of diarrhea in the preceding 2 wk served as an exclusion factor. **Written consent was obtained from adult family members.

Viral RNA was purified from ≈200 mg fecal specimen and 140 µL nasal swab specimen suspended in phosphate-buffered saline by using the Viral RNA Mini kit (QIAGEN, São Paulo, Brazil) as described (*7*). A nested reverse transcription PCR (*3*) detected cosavirus. After nucleotide sequencing of all PCR-positive specimens, we developed a specific real-time RT-PCR for quantifying viruses from Brazil (Table 2). Assay optimization and quantification relied on photometrically quantified cRNA in vitro transcripts, as described (*8*). After assay optimization, sensitivity was 6.8 copies per reaction.

**Table 2:** Oligonucleotides used for detection and quantification of cosaviruses*

Oligonucleotide identity	Sequence, 5′ → 3′	Genomic target region, RT-PCR type	Use	Reference
DKV-N5U-F1	CGTGCTTTACACGGTTTTTGA (+)	5’-UTR, nested RT-PCR 1st round	Cosavirus detection†	(*3*)
DKV-N5U-R2	GGTACCTTCAGGACATCTTTGG (–)			
DKV-N5U-F2	ACGGTTTTTGAACCCCACAC (+)	5’-UTR, nested RT-PCR 2nd round		
DKV-N5U-R3	GTCCTTTCGGACAGGGCTTT (–)			
HCosV-rtF735-1	TTGTAGYGATGCTGTRTGTGTGTG (+)	5’-UTR, real time RT-PCR	Brazilian cosavirus quantification†	This study
HCosV-rtP783	FAM-AGCCTCACAGGCCRRAAGCCCTGTC-DDQ1 (+, Probe)			
HCosV-rtR827-1	CCAYTGTGTGGGTCCTTTCG (–)			

*RT-PCR, reverse transcription PCR; UTR, untranslated region; FAM, fluorescein; R, G/A; DDQ1, deep dark quencher 1; Y, C/T. †RT-PCR reactions were carried out using the QIAGEN One-step RT-PCR kit as described by the manufacturer (QIAGEN, São Paulo, Brazil), 300 nmol/L of each primer, 200 nmol/L of the probe (real time RT-PCR assay), 1 μg bovine serum albumin, and 5 μL RNA extract. Second-round reactions used the Platinum Taq DNA Polymerase Kit as described by the manufacturer (Invitrogen, São Paulo, Brazil) with 2.5 mol/L MgCl and 1 µL of first-round PCR product. Real time RT-PCR amplification involved 55°C for 15 min, 95°C for 15 min, and 45 cycles of 95°C for 15 s and 58°C for 30 s (fluorescence measured).Nested RT-PCR involved 30 min at 50°C; 15 min at 95°C; 10 cycles of 20 s at 94°C, 30 s starting at 60°C with a decrease of 1°C per cycle, and 50 s at 72°C; and 40 cycles of 20 s at 95°C, 30 s at 54°C, and 50 s at 72°C; and a final elongation step of 5 min at 72°C. Second-round reactions used 3 min at 94°C and thermal cycling as for the first round.

In the adult cohorts, 1 of 105 HIV-infected persons with gastroenteritis had positive results for cosavirus. In the control group of 49 HIV-infected adults without gastroenteritis, none had positive cosavirus results (χ^2^ 0.5; p = 0.49).

Considerably higher prevalence was detected among child cohorts. Of children with gastroenteritis, 13 (3.6%) of 359 patients were cosavirus positive. The proportion of cosavirus-positive controls without gastroenteritis, sampled in 2008, was significantly higher: 65 (49.2%) of 132 controls were cosavirus positive (χ^2^ 149.1; p<0.0001). On resampling in 2011, 4 (6.5%) of 62 controls were cosavirus positive. Although the difference was not statistically significant (χ^2^ 1.1; p = 0.3), this result was almost double that for patients. In another child-care center, none of 10 healthy children were cosavirus positive.

**Figure 1:**
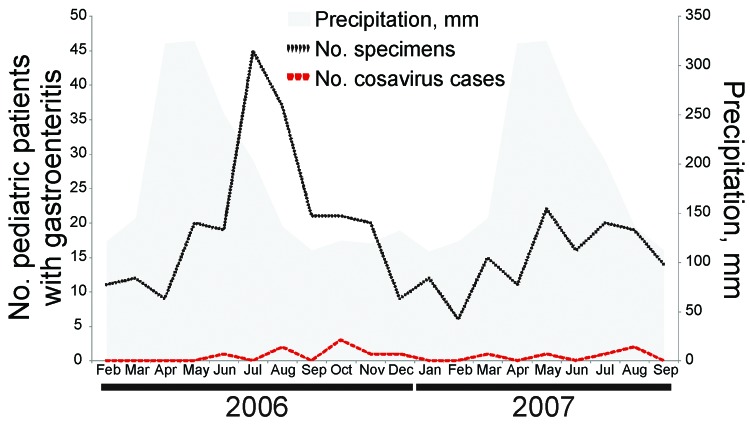
Detection pattern of cosavirus in children with gastroenteritis throughout different seasons during 2006–2007, Brazil. Temperature was not plotted because it varied little from mean 25.2°C through the year (range 23.6–26.7°C). Precipitation data were obtained from the German Weather Service and represent means throughout 1961–1990.

The higher prevalence detected in controls in 2008 could indicate a seasonal infection pattern because specimens were collected in a single weekend in a child-care center. The lower cosavirus-positive results in the same child-care center 3 years after the initial sampling support this idea. However, in sick children, cosavirus-positive specimens were obtained at similarly low rates throughout the year (Figure 1), which might argue against seasonal variation as a generic property of cosavirus infection.

**Figure 2:**
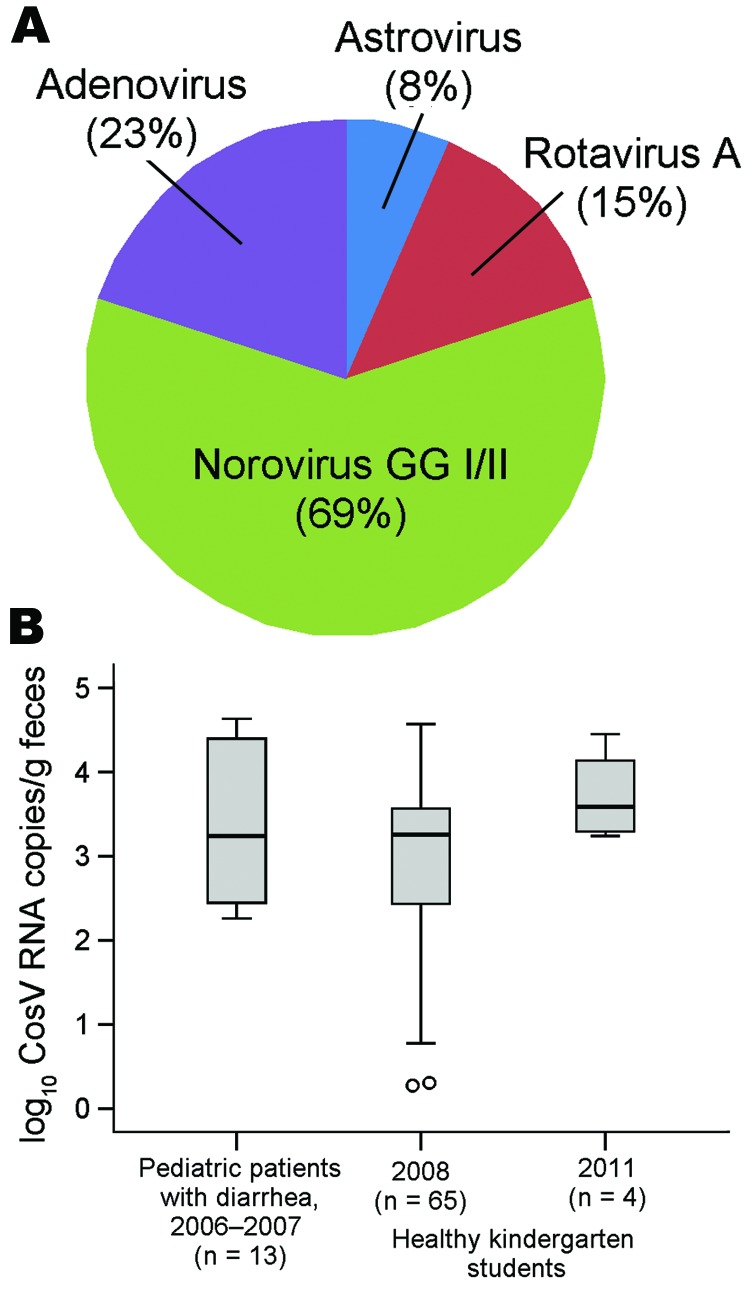
Co-infections and fecal cosavirus (CosV) RNA concentrations. A) Co-infections with established viral causes of diarrhea in children with gastroenteritis who were positive for CosV. Viral RNA and DNA were detected by real-time PCR (methods available upon request) in the same eluates used for CosV detection. B) Boxplot generated with SPSS V19 (SPSS, Munich, Germany) of log_10_ CosV RNA concentrations per gram of feces in children with gastroenteritis and healthy control children from a child-care center in 2008 and 2011. Boxes show the medians and interquartile ranges (box length). The whiskers represent an extension of the 25th or 75th percentiles by 1.5× the interquartile range. Datum points beyond the whisker range are considered as outliers and marked as circles. GGI/II, genogroups I and II.

To evaluate whether cosavirus causes disease in ill children, we analyzed co-infections with common viral pathogens causing diarrhea (astrovirus, norovirus, rotavirus, adenovirus) (Figure 2, panel A). In 10 (76.9%) of 13 cosavirus-positive patients, >1 of these pathogens could be detected.

Acute infection with an enteric virus is usually associated with high viral RNA shedding. Cosavirus concentrations were low in outpatient and control children (≈10^3^ copies/g) on 2 sampling occasions (Table 1) and did not differ significantly (analysis of variance *F* 2.0; p = 0.14; Figure 2, panel B).

Low cosavirus concentrations in the enteric tract could result from swallowing viruses originating in the respiratory tract. Therefore, we tested for cosavirus in 96 nasal swab specimens from gastroenteritis patients, including 3 nasal swab specimens from persons with cosavirus-positive fecal specimens and 65 nasal swab specimens from cosavirus-positive controls sampled in 2008. None of the nasal specimens from controls with positive fecal specimens and only 1 nasal specimen from a patient unrelated to the 3 aforementioned persons was weakly positive (10^3^ RNA copies/mL), which refutes this hypothesis.

To analyze whether a preceding point-source infection caused high cosavirus prevalence in the controls without gastroenteritis sampled in 2008, we determined the genomic sequence of the 5′ untranslated region PCR amplicons and phylogenetically analyzed the sequence (GenBank accession no. JN228118–JN228188). Cosaviruses from these controls were distributed across the phylogenetic tree (Technical Appendix). Maximum nucleotide distance within these cosaviruses was up to 22.5% in the analyzed 398-nt fragment, making a recent point-source infection unlikely.

## Conclusions

Human cosavirus infections were reported previously from a limited number of persons and geographic areas (*3**–**6*). In Brazil, the 3.6% detection rate in children with gastroenteritis was comparable to the 1.8% rate in a cohort study of gastroenteritis patients in China (*6*). Although the 6.5% detection rate in 1 control cohort in Brazil was compatible with the 1.7% rate in 60 healthy controls in China, the combined 33.8% prevalence detected in controls from 3 different samplings in Brazil was much higher. Nonetheless, the prevalence was comparable to the 43.9% detected in 41 healthy Southeast Asian children in the only other cohort study (*3*). Detecting cosavirus in 1 of 154 adults in Brazil was compatible with finding a single cosavirus-positive patient among 1,000 adults with gastroenteritis in Scotland, confirming that cosaviruses are rare and probably neither pathogenic nor commensal in adults (*3*).

The higher prevalence of cosavirus found in controls than in patients, the frequent co-infections with established pathogens, and the unusually low RNA virus concentrations give evidence against cosavirus involvement in human gastroenteritis. Viruses that replicate in the human gut generally reach concentrations 1,000- to 100,000-fold higher than those of cosavirus. This finding is exemplified by genetically related picornaviruses (Aichi viruses, parechoviruses, and cardioviruses) and established enteric pathogens (e.g., noroviruses and rotaviruses) (*8**–**12*). Notably, the aforementioned study on cardioviruses included the same specimens from Brazil, which indicates that poor sample quality was not a factor.

These low concentrations would be compatible with absence of replication in the enteric tract and passive virus ingestion, e.g., from nutritional sources, drinking water, or the respiratory tract. However, nutritional patterns of the tropical countries in which cosavirus have been detected certainly differ. Furthermore, in Brazil, adults are unlikely to have a completely different diet from infants and children. Moreover, the unprecedented detection of cosavirus in a respiratory tract specimen makes ingestion of viruses from nutritional sources alone unlikely, although a link to fluid droplets from drinking water in the respiratory tract is hypothetically possible.

Another explanation for low cosavirus RNA levels in fecal samples is that a cosavirus infection occurred early in the person’s life and produced partial mucosal immunity and limited subsequent cosavirus replication in the gut. This is exemplified for viruses transmitted by the fecal–oral route by up to 100-fold higher fecal shedding of vaccine rotavirus and poliovirus among seronegative persons than among seropositive or previously vaccinated persons (*13**,**14*). However, this explanation would be incompatible with the high prevalence of cosavirus in many control children, who were generally older than patients.

Prolonged low concentrations of picornavirus shedding has been demonstrated, e.g., by detectable hepatitis A virus RNA up to 3 months after acute infection (*15*). Nonetheless, this circumstance is unlikely to explain the low cosavirus concentrations, given the overall high number of persons with positive results.

Although our study extends the known geographic occurrence of cosavirus, whether it is a human pathogen remains to be determined. Future studies would be enhanced by serologic analyses and investigations focusing on nutrition and drinking water in tropical countries.

## Supplementary Material

Technical Appendix Cosavirus (CosV) partial 5′ untranslated region phylogeny.
