# Supplementary material for: Cosavirus Infection in Persons with and without Gastroenteritis, Brazil
Source: Emerg Infect Dis. 2012 Apr;18(4):656–9. doi: 10.3201/eid1804.111415 (PMC3309695; doi:10.3201/eid1804.111415)
Supplement: Technical Appendix — Cosavirus (CosV) partial 5′ untranslated region phylogeny. [file 11-1415-Techapp_1p.pdf]

Article DOI: http://dx.doi.org/10.3201/eid1804.111415

Cosavirus Infection in Persons with and without Gastroenteritis, Brazil

Technical Appendix

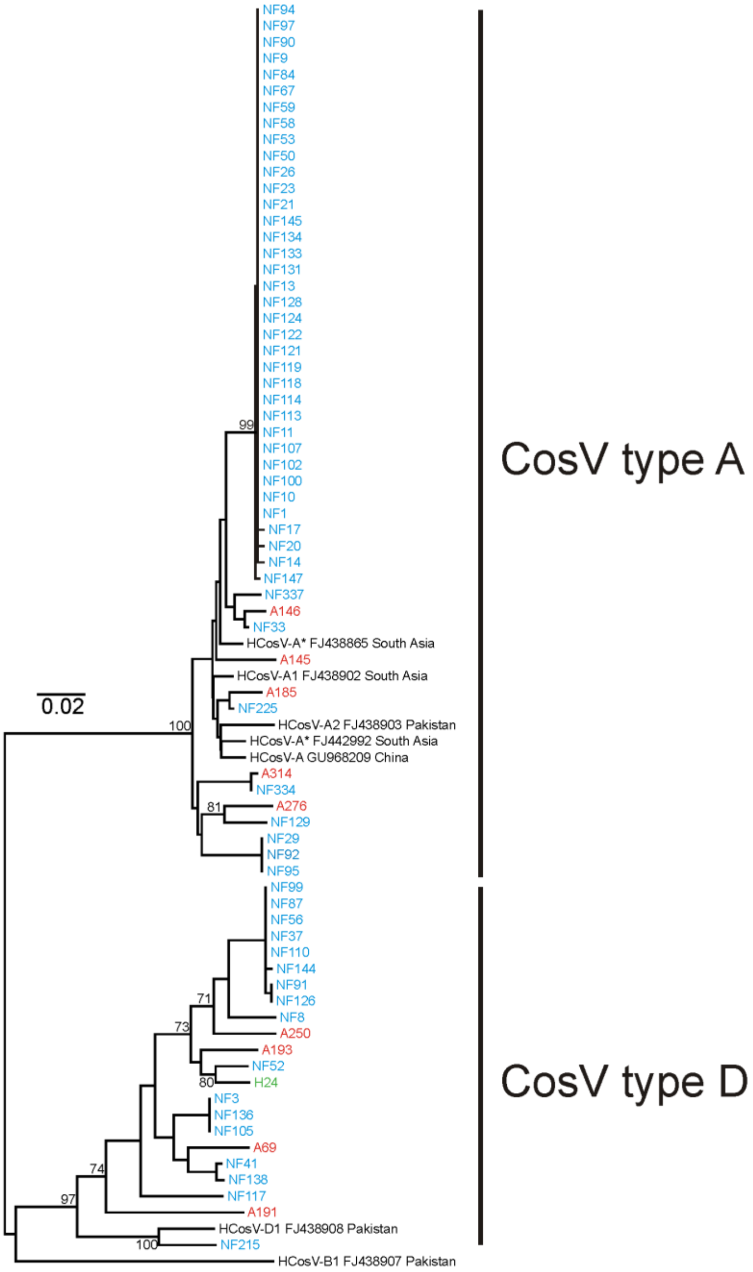

Technical Appendix Figure. Cosavirus (CosV) partial 5′ untranslated region phylogeny. The neighbor-joining phylogeny was generated by using MEGA5 (www.megasoftware.net), including a complete deletion option and a nucleotide percentage distance substitution model drawn to scale. Values at node points indicate support from 1,000 bootstrap reiterations (only values >70 are shown). The final dataset comprised 389 t corresponding to positions 512–909 in CosV A1 (GenBank accession no. FJ438902). All available CosV sequences covering the complete genomic region obtained in this study were included in the analysis and are given with strain name (when available), GenBank accession number and geographic origin. CosV 5′ untranslated region types (*3*) are indicated to the right of taxon names. Viruses from this study are color coded by clinical cohort: red, children with gastroenteritis; green, HIV-infected adults with gastroenteritis; blue, healthy control children from child-care centers. Scale bar indicates nucleotide substitutions per site.
